# Supplementary material for: Molecular basis for DNA recognition by the maternal pioneer transcription factor FoxH1
Source: Nat Commun. 2022 Nov 26;13:7279. doi: 10.1038/s41467-022-34925-y (PMC9701222; doi:10.1038/s41467-022-34925-y)
Supplement: Supplementary file 1 — Supplementary Information [file 41467_2022_34925_MOESM1_ESM.pdf]

Supplementary material for

Molecular basis for DNA recognition by the maternal pioneer transcription factor FoxH1

Authors

Radoslaw Pluta[1,8]; Eric Aragón[1,8]; Nicholas A. Prescott[2,3]; Lidia Ruiz[1], Rebeca A. Mees[1], Blazej Baginski[1]; Julia R. Flood[3]; Pau Martin-Malpartida[1]; Joan Massagué[4]; Yael David[3,5-6] and Maria J. Macias[1,7*]

Affiliations

1. Institute for Research in Biomedicine (IRB Barcelona), The Barcelona Institute of Science and Technology (BIST), Barcelona 08028, Spain;
2. Tri-Institutional PhD Program in Chemical Biology, New York, NY, USA;
3. Chemical Biology Program, Memorial Sloan Kettering Cancer Center, New York, NY 10065, USA;
4. Cancer Biology and Genetics Program, Memorial Sloan Kettering Cancer Center, New York, NY 10065, USA;
5. Department of Pharmacology, Weill Cornell Medicine, New York, NY 10065, USA;
6. Department of Physiology, Biophysics and Systems Biology, Weill Cornell Medicine, New York, NY 10065, USA;
7. Institució Catalana de Recerca i Estudis Avançats (ICREA), Passeig Lluís Companys 23, Barcelona 08010, Spain.
8. These authors contributed equally to this work

*Corresponding author: maria.macias@irbbarcelona.org

Table of Contents

Figures and Tables 3

Supplementary Figure 1 3
Supplementary Figure 2 5
Supplementary Figure 3 6
Supplementary Figure 4 7
Supplementary Figure 5 8
Supplementary Figure 6 10
Supplementary Figure 7 12
Supplementary Table 1 13
Supplementary Table 2 14
Supplementary Table 3 15
Supplementary Table 4 16
Supplementary Table 5 19

Synthetic DNA sequences 21
Mutation primers 22
Supplementary References 23

Figures and Tables

Supplementary Figure 1

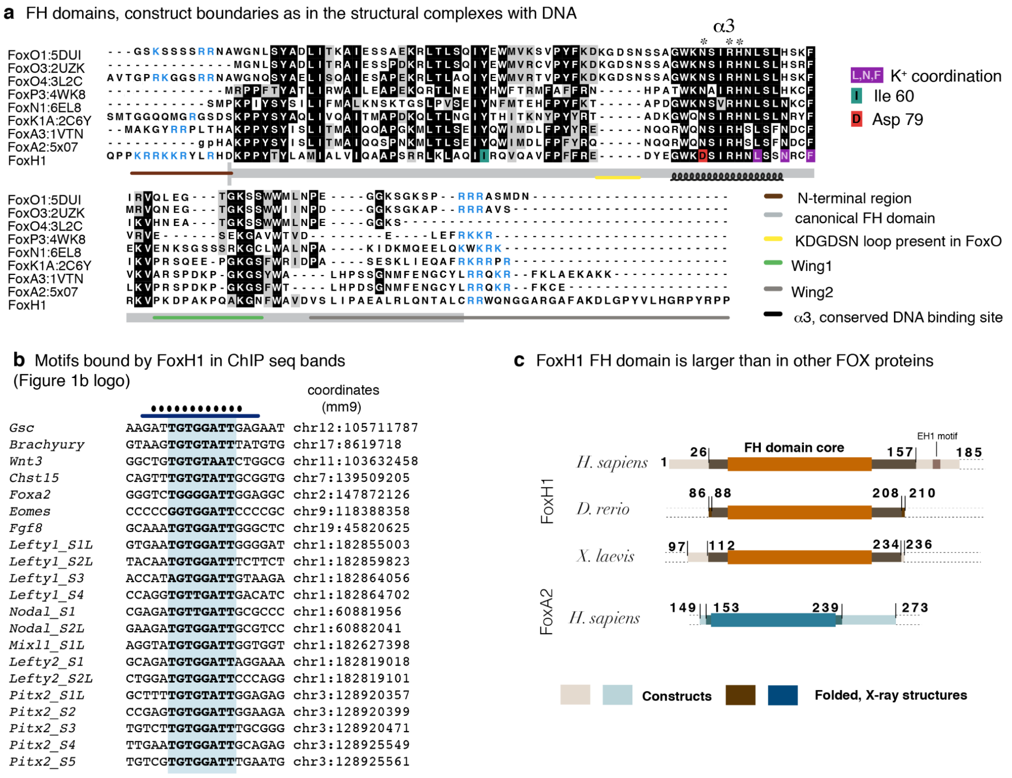

a. Sequence alignment of different FH domains whose structures have been characterized. PDB entries are included next to the protein name.

b. Selected peaks bound by the FoxH1 protein in ChIP-seq assays (GSE125116, [1]. The canonical motif and base pairs of *Gsc* that participate in specific contacts with the protein are highlighted. Coordinates and nucleosome positions are indicated (as described in GSM2842982).

c. Constructs used for the structural studies indicating the expressed proteins and the region observed in the X-ray structures. The human sequence contains the TLE binding site, but this region does not contribute to the extended FH domain structure.

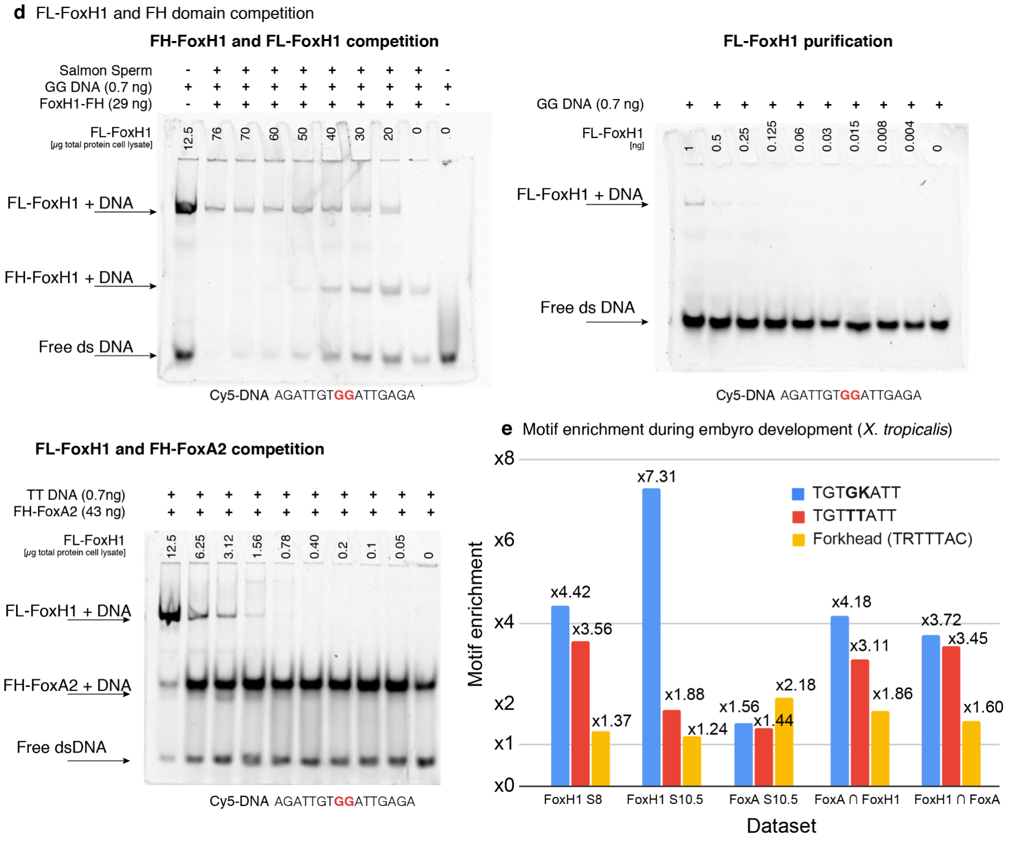

d. Left: Competition assay between FL-FoxH1 (cell lysate) and the recombinant FH domain used in the structural work binding to the GG site. Right: Purified FL-FoxH1 from cell lysates using anti-FLAG®M2 Affinity Gel (Sigma) and eluted by competition with 3X FLAG® Peptide (Sigma). Bottom: Competition between the FH-FoxA2 domain and FL-FoxH1 (cell lysate) using the forkhead site.

e. Motif enrichment observed in ChIP-seq datasets (Methods section) during embryo development. Motifs used in the search are indicated in the figure. The last two sets correspond to the intersection of FoxA and FoxH1 peaks at stages 10.5 and 8 respectively, when both proteins are present at high concentrations.

Supplementary Figure 2

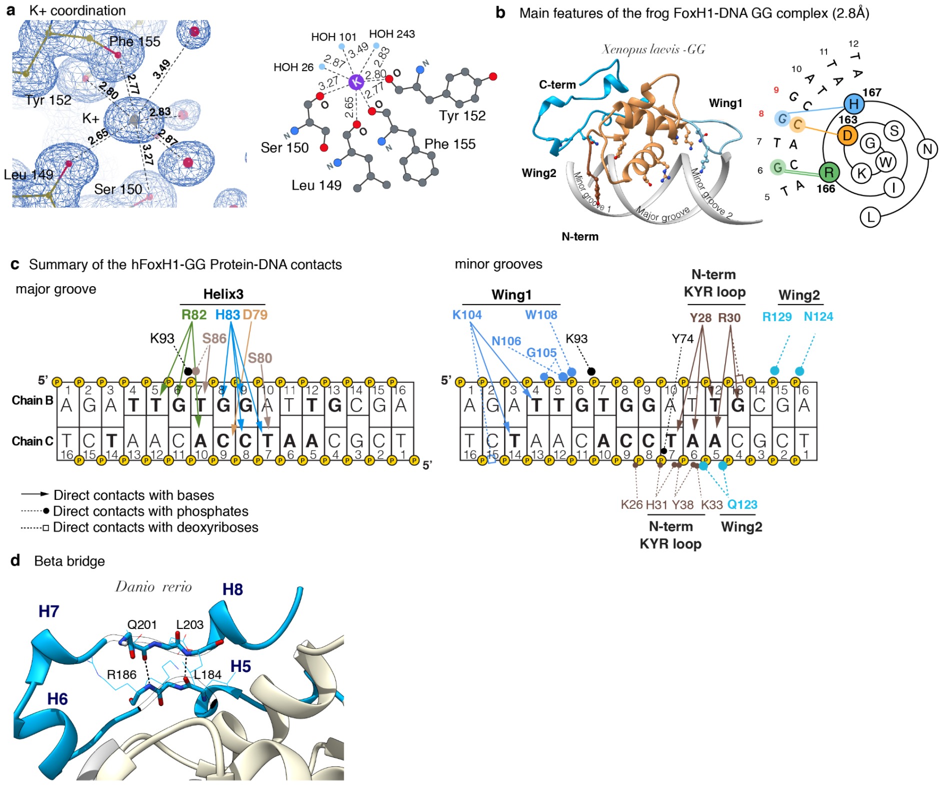

a. The potassium cation (K^+) is coordinated by four carbonyl oxygen atoms from the polypeptide backbone (Leu149, Ser 150, Tyr 152 and Phe 155 and three well-ordered water molecules. The ion is indicated as a violet sphere (Ligplot+ V2.2) [2].

b. Structure of the *Xenopus laevis* FoxH1-DNA complex bound to the GG motif (crystallographic asymmetric unit). 2D Wenxiang diagram (H3) showing the side chains and specific base contacts with the major groove.

c. Specific contacts for the human complex refined at 1.5Å resolution displaying the 15 bp recognition through a rich network of direct contacts separated in two plots (major and minor groove interactions) for clarity. Residue colors as in panel B.

d. Snapshots showing the high-resolution electron-density maps of the GG and GT complexes for regions covering the H3 helix. 2Fo-Fc maps are contoured at 1.0 sigma. Snapshots highlighting direct and water-mediated polar contacts with nucleobases (distance up to 3.6 Å). The side chain orientation and the contacts are almost identical in the two complexes.

Supplementary Figure 3

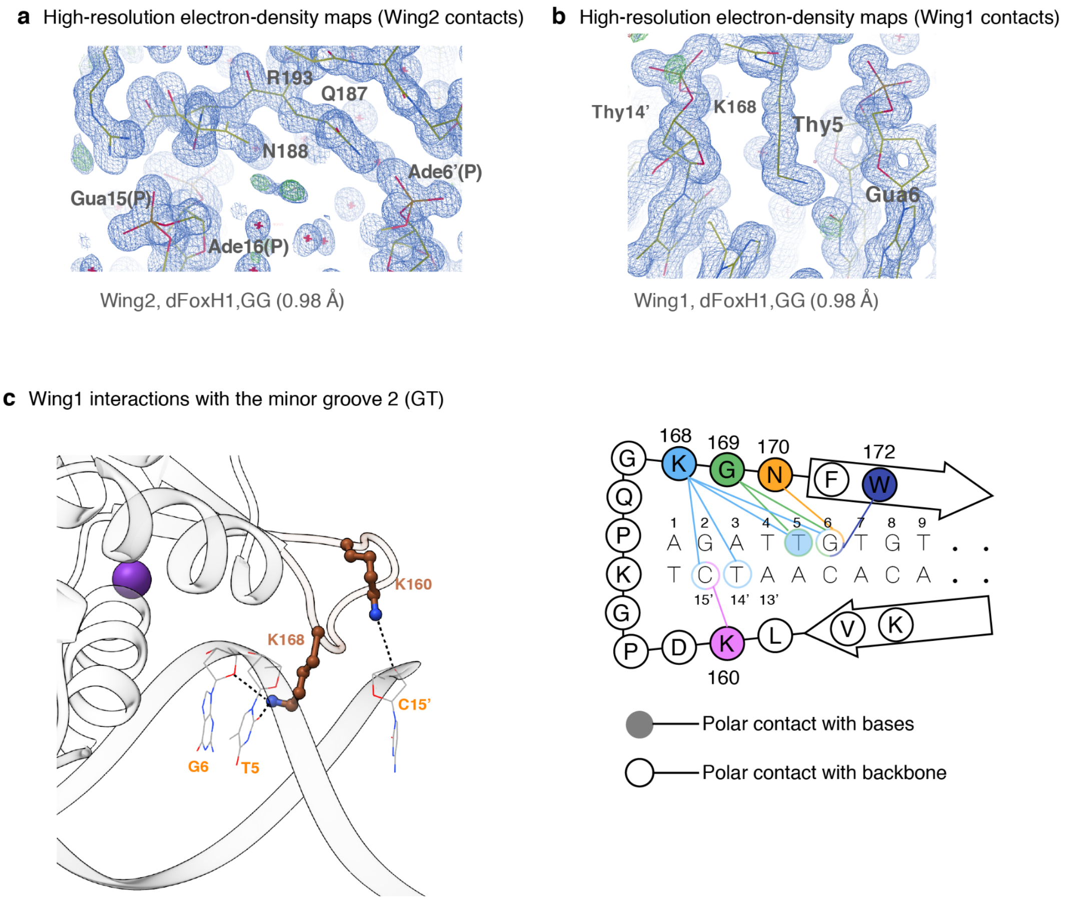

a. Wing 2 contacts: 2Fo-Fc map contoured at 1.0 sigma for the GG complex refined at 0.98Å

b. Wing 1 contacts: 2Fo-Fc map as in a.

c. DNA contacts observed from Wing1 to minor groove 2. HBs from the lysine residues are shown in the cartoon representation of the structure. Polar contacts are represented schematically on the adjacent panel.

Supplementary Figure 4

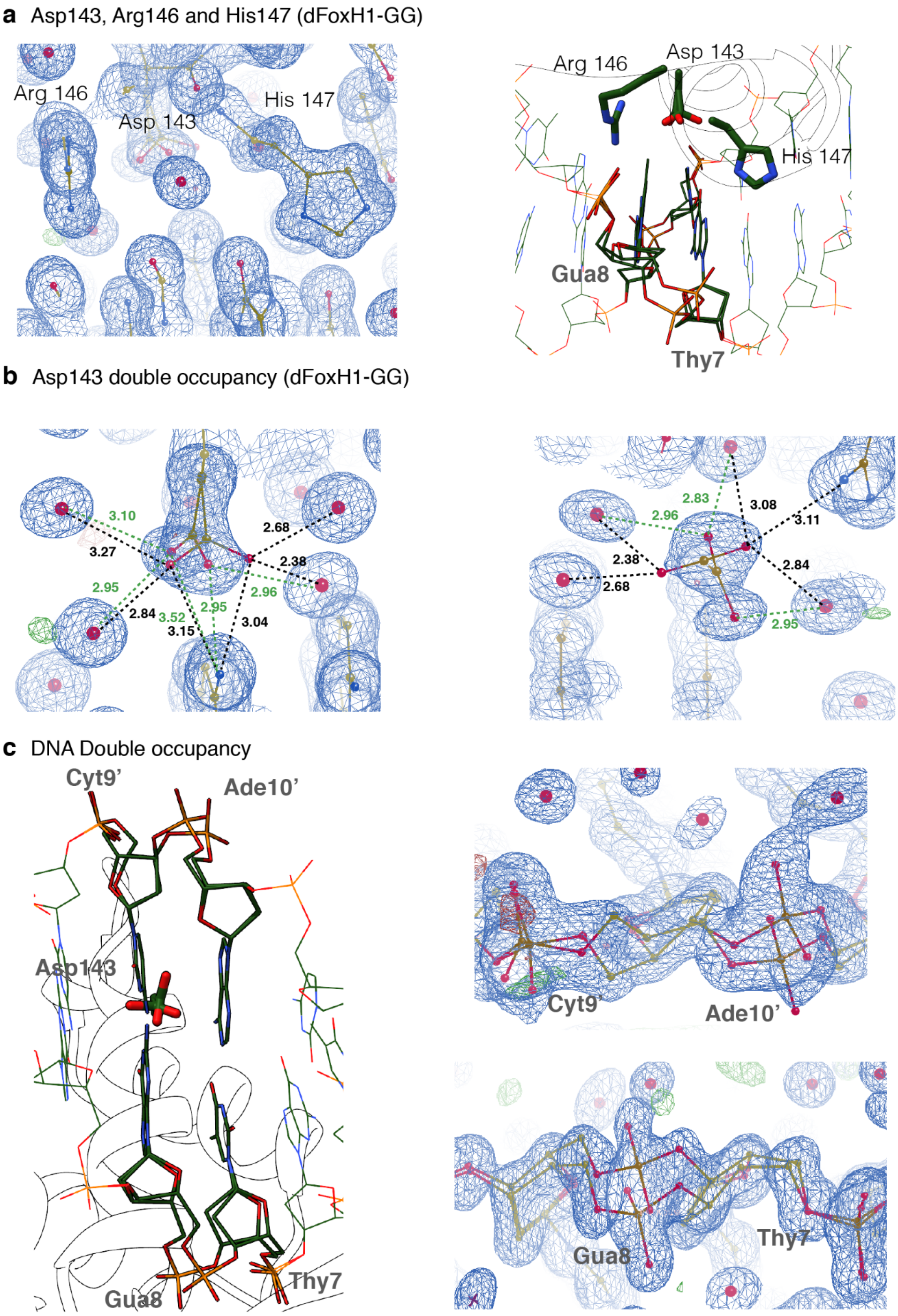

a. 2Fo-Fc map contoured at 1.0 sigma for the dFoxH1-GG complex refined at 0.98Å, showing the Helix3-DNA contacts

b. The orientations observed for the Asp side chain and the DNA in the GG complex are highlighted. Distances are in green for one orientation and in black for the other.

c. Double occupancy of the DNA at the Asp interaction site.

Supplementary Figure 5

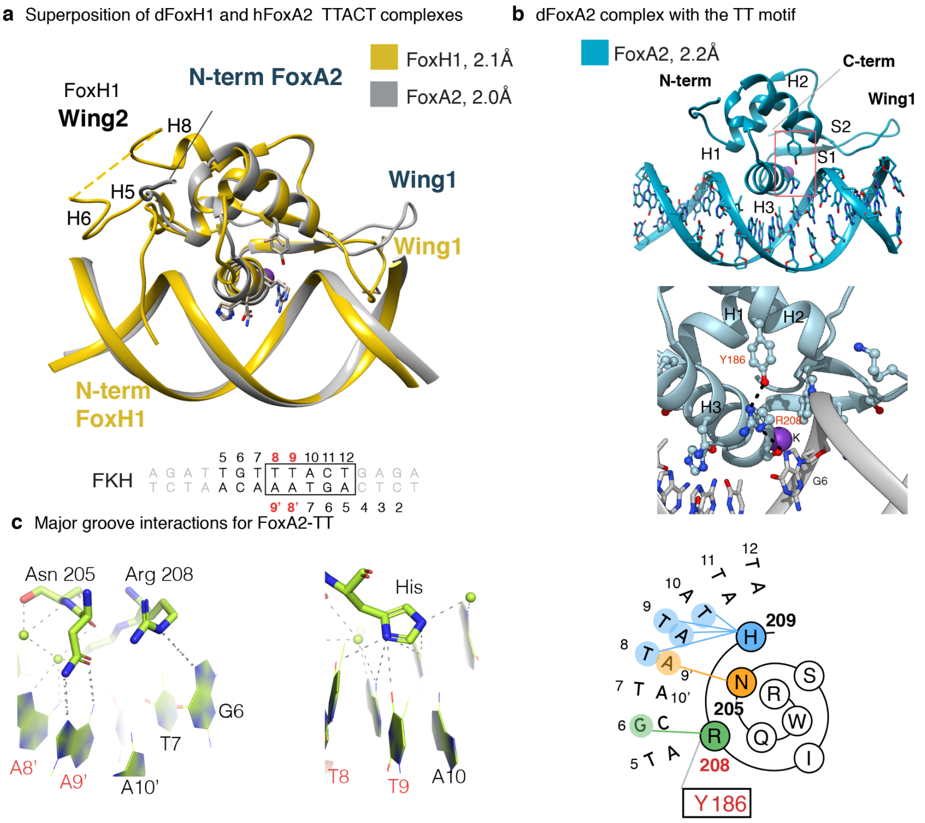

a. Comparison of FoxH1 (gold) and FoxA2 (argent) binding to the canonical forkhead site determined in this work. Wing2 in FoxH1 is less ordered than in the GG and GT complexes, but the contributions to DNA binding are still observed for both Wing regions and the N-terminal loop. Although the FoxA2 construct used for the crystal structure is as long as that of FoxH1, FoxA2 does not have a folded Wing2 and neither Wing1 nor the N-term interact specifically with the minor grooves 1 and 2 as observed in FoxH1 complexes. The FoxA2 complex was refined at 2.0 Å resolution respectively (Table 1)

b. We also determined FoxA2 bound to the TT site at 2.2 Å resolution, shown in cyan. The orientation of the Arg side chain is away from the major groove, as observed in the other FOX complexes.

c. Snapshots highlighting direct and water-mediated polar contacts from the Asn205, Arg208 and His209, located in H3, with nucleobases (distance up to 3.6 Å) of the major groove. A summary of the FoxA2 interactions with the TT site are represented as a 2D Wenxiang diagram

d DNA groove parameters, FoxH1 complexes

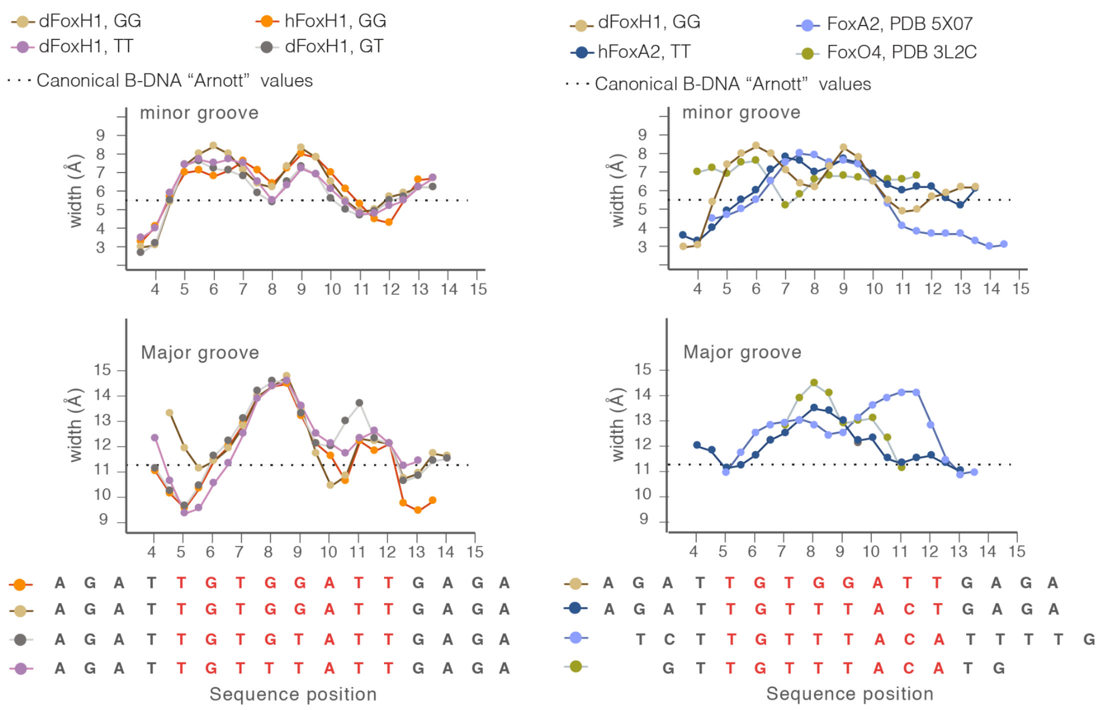

e Hoogsteen (GT and TT) base-pairs

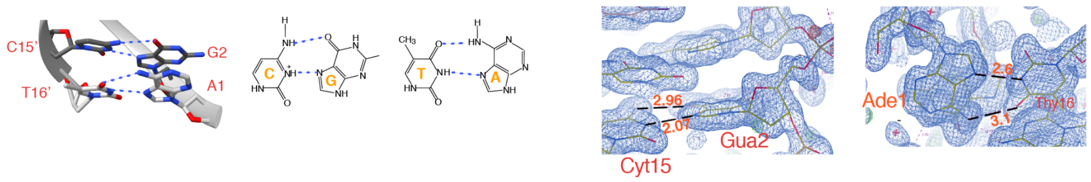

d. Analyses of minor and major groove widths of the different FoxH1 complexes. Comparison of the minor and major groove width values of FoxH1 GG complex (orange) to FoxA2 and FoxO4 structures (light and dark blue). All values were calculated using Curves+ [3].

e. Hoogsteen base-pairs observed in the dFoxH1-GG structure at the GT and TT bps. Two snapshots of the 2Fo-Fc map contoured at 1.0 sigma showing the crystal environment surrounding the two bps which form HG bps (Ade1:Thy16 and Gua2: Cyt15).

Supplementary Figure 6

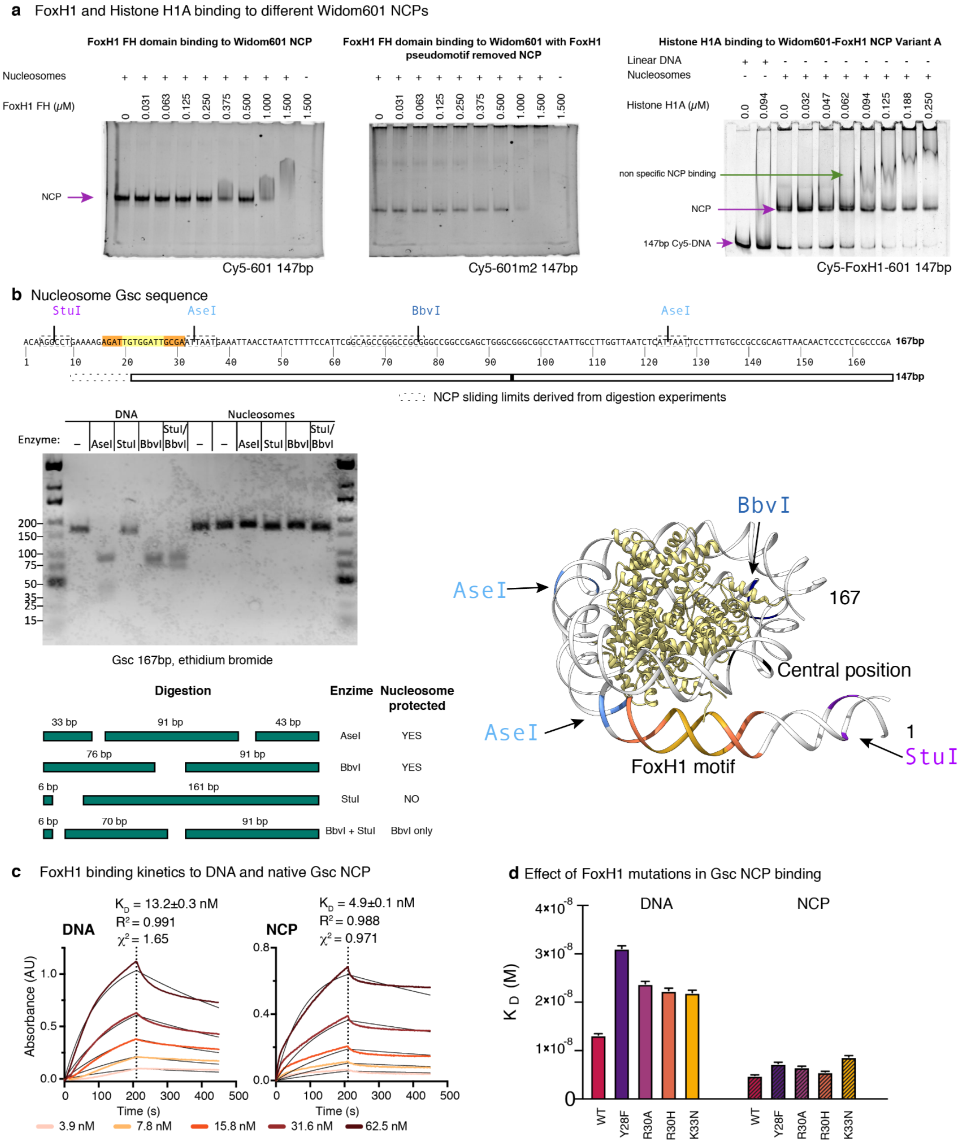

a. Titration of the Widom601 and Widom601m2 NCPs with the FoxH1 FH domain followed by EMSAs. Sequence-agnostic binding of the Histone H1A FH domain and the A variant of the 147 bp NCP. Arrows indicate the different species

b. Enzymatic digestion of the 167 NCP using three enzymes that specifically cut the linear DNA sequence. Only StuI (whose site is located at the 5' end) is able to cut it when the nucleosome is formed. This result indicates that the StuI site is exposed whereas the other sites are protected. We have illustrated the most extreme case, where the nucleosome is formed using the remaining bps, to

display the FoxH1 site, which is still located in the supercoiled area of the NCP. The three enzyme-cutting sites are also indicated in the model.

c. Binding kinetics between the FoxH1 FH domain and either free *Gsc*167 or nucleosomes, as shown by Biolayer Interferometry.

d. Effect of point mutations of the KYR loop on DNA binding affinity. Native *Gsc* NCP and the GG site were used in these experiments. All WT and FoxH1 mutated proteins bind well to DNA and NCP. Bars represent fitted K_D values and error bars represent fit uncertainty. Source Data are provided.

Supplementary Figure 7

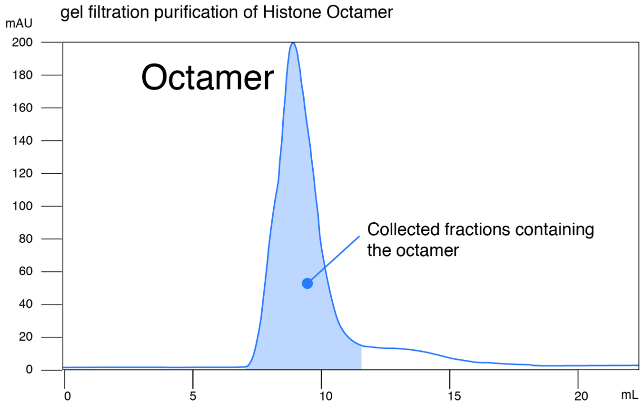

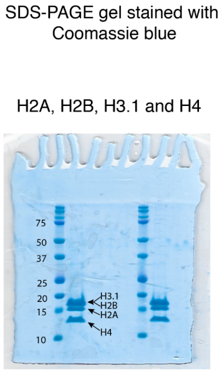

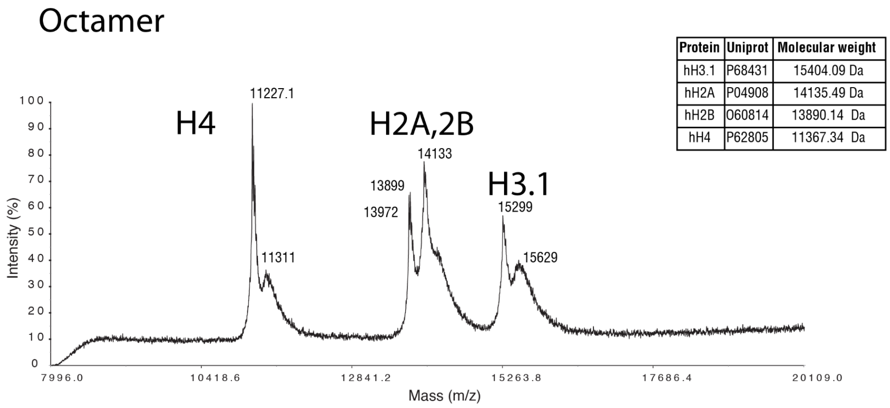

Protein	Uniprot	Molecular weight
hH3.1	P68431	15404.09 Da
hH2A	P04908	14135.49 Da
hH2B	O60814	13890.14 Da
hH4	P62805	11367.34 Da

Mass and purity of the histones used for NCP preparation.

Supplementary Table 1

FoxH1 and FoxA2 melting temperatures and DNA stabilization.

**H-FoxH1 free**	44.9 ± 0.2 °C	**X-FoxH1 free**	40.3 ± 0.1 °C
**H-FoxH1 + GG DNA**	62.1 ± 0.1 °C	**X-FoxH1 + GG DNA**	50.6 ± 0.1 °C
**H-FoxH1 + TT DNA**	59.3 ± 0.1 °C	**D-FoxH1ΔWing2 free**	40.5 ± 0.1 °C
**H-FoxH1 + GT DNA**	61.1 ± 0.1 °C	**D-FoxH1ΔWing2 +GG DNA**	40.2 ± 0.6 °C
**H-FoxH1 + TTACT DNA**	55.5 ± 0.1 °C	**H-FoxA2 free**	46.6 ± 0.3 °C
**D-FoxH1 free**	40.7 ± 0.4 °C	**H-FoxA2 + GG DNA**	44.7 ± 0.2 °C
**D-FoxH1 + GG DNA**	60.8 ± 0.3 °C	**H-FoxA2 + TT DNA**	54.4 ± 0.5 °C
**D-FoxH1 + TT DNA**	59.9 ± 0.2 °C	**H-FoxA2 + GT DNA**	50.8 ± 0.1 °C
**D-FoxH1 + GT DNA**	61.1 ± 0.2 °C	**H-FoxA2 + TTACT DNA**	56.4 ± 0.3 °C
**D-FoxH1 + TTACT DNA**	57.4 ± 0.3 °C		

H- *Homo sapiens*, D- *Danio rerio*, X- *Xenopus laevis*.

Melting temperatures are indicated as mean ± s.d (n=4).

Supplementary Table 2

Oligonucleotides used for crystallization of FoxH1 and FoxA2 DBD-DNA complexes (canonical FoxH1 and forkhead motifs underlined).

Structure	Oligonucleotides	dsDNA name
**xFoxH1-GG**	5'-CAGATTGTGGATTGAG-3'<br>5'-CTCAATCCACAATCTG-3'	dGSC-GG-16b
**hFoxH1-GG**	5'-AGATTGTGGATTGCGA-3'<br>5'-TCGCAATCCACAATCT-3'	hGSC-GG-16a
**dFoxH1-GG**	5'-AGATTGTGGATTGAGA-3'<br>5'-TCTCAATCCACAATCT-3'	dGSC-GG-16a
**dFoxH1-GT**	5'-AGATTGTGTATTGAGA-3'<br>5'-TCTCAATACACAATCT-3'	dGSC-GT-16a
**dFoxH1-TT**	5'-AGATTGTTTATTGAGA-3'<br>5'-TCTCAATAAACAATCT-3'	dGSC-TT-16a
**dFoxH1-TTACT**	5'-AGATTGTTTACTGAGA-3'<br>5'-TCTCAGTAAACAATCT-3'	FKH-TTACT-GSCflank
**hFoxA2-TT**	5'-AGATTGTTTATTGAGA-3'<br>5'-TCTCAATAAACAATCT-3'	dGSC-TT-16a
**hFoxA2-TTACT**	5'-AGATTGTTTACTGAGA-3'<br>5'-TCTCAGTAAACAATCT-3'	FKH-TTACT-GSCflank

Supplementary Table 3

Quantitative analysis of protein-DNA interactions and interfaces in FoxH1 and other complexes (DNAproDB web tool).

Protein	DNA	PDB	Res. (Å)	Interactions*	BASA [Å²]	HBs Total	VdW Total
**hFoxH1**	TGTGGATT	7YZB	1.47	73	1581	31	158
**dFoxH1**	TGTGGATT	7YZ7	0.98	66	1481	31	146
**dFoxH1**	TGTGTATT	7YZA	1.18	74	1667	35	162
**dFoxH1**	TGTTTATT	7YZC	2.17	71	1553	28	154
**dFoxH1**	TGTTTACT	7YZD	2.13	71	1470	24	164
**xFoxH1**	TGTGGATT	7YZG	2.80	57	1346	24	109
**hFoxA2**	TGTTTACT	7YZE	1.99	42	1012	13	72
**hFoxA2**	TGTTTATT	7YZF	2.18	42	904	11	59
**Zfp57**	TGCCGC	4gzn	0.99	53	1164	29	114
**FoxA3**	GGTTGAC	1vtn	2.50	48	1181	15	102
**FoxA2**	TGTTTAC	5x07	2.80	39	941	12	66
**FoxK2**	TGTTTAC	2c6y	2.40	48	1240	16	85
**FoxG1**	TGTTTAC	7cby	1.65	44	1009	14	70
**FoxC2**	TGTTTAC	6ako	2.40	41	1053	14	82
**FoxC2**	TGTTTAT	6o3t	3.10	42	888	14	80
**FoxN1**	GCGTC	6el8	1.61	37	818	13	74
**FoxN3**	GCGTC	6ncm	2.70	41	815	12	70
**FoxN3**	TGTTTAC	6nce	2.60	38	861	9	60
**FoxM1**	TGTTTAT	3g73	2.20	36	850	14	52
**FoxO1**	TGTTTTG	3coa	2.20	37	847	13	61
**FoxO1**	TGTTTAC	3co7	2.90	36	885	13	57
**FoxO1**	TGTTTAC	3co6	2.10	39	856	14	62
**FoxO4**	TGTTTAC	3l2c	1.87	39	831	18	71
**FoxO3**	TGTTTAC	2uzk	2.70	40	988	11	72

*Residue-nucleotide interactions. BASA: buried solvent accessible surface area

Supplementary Table 4

List of reagents.

REAGENT or RESOURCE	SOURCE	IDENTIFIER
DNase I	Roche	21864900
Phusion polymerase	Thermo Scientific	2F-530S
Lysozyme	FLUKA	101174393
PMSF	BIO BASIC CANADA	PB0425
SigmaFast	Merck & Co., Inc.	28830-TAB
Ampicillin	Melford	A40040
SYPRO Orange Protein gel stain	Merck & Co., Inc.	S5692
Glycine	Melford	G0709
TRIS	Melford	B2005
Bis-TRIS	Melford	B75000
LB	Melford	L24060
IPTG	Melford	I56000
Tween-20	Merck & Co., Inc	P2287
Glycerol	Merck & Co., Inc	49767
BSA	Life Technologies	AM2616
NaCl	LabKem	SOCH-001-5K0
$MgCl_2$	Merck & Co., Inc	8147330100
$ZnCl_2$	Merck & Co., Inc	793523-100G
KCl	Merck & Co., Inc	529552
Ammonium acetate	Merck & Co., Inc	09689
Sodium acetate	Merck & Co., Inc	241245
NH_4Cl	Merck & Co., Inc	213330
Ethylene glycol	Merck & Co., Inc	324558
Poly(ethylene glycol) 3350	Molecular Dimensions	MD2-100-9
Poly(ethylene glycol) 8000	Molecular Dimensions	MD2-100-13
PEG Smear Low	Molecular Dimensions	MD2-100-258

PEG Smear High	Molecular Dimensions	MD2-100-260
Poly(ethylene glycol) 400	Molecular Dimensions	MD2-250-3
Lithium sulfate	Molecular Dimensions	MD2-100-45
Sodium cacodylate	Molecular Dimensions	MD2-021_ 2M
EDTA	Merck & Co., Inc.	EDS
DTT	Life Technologies	R0861
Guanidine HCl	Merck & Co., Inc.	G3272
Acrylamide 40%, 19:1	Panreac	A3658,0500
Sucrose	Merck & Co., Inc.	S7903
Ethidium Bromide	Fisher Scientific	11508756
TCEP	Merck & Co., Inc.	C4706
Salmon sperm dsDNA	Merck & Co., Inc.	D1626
Orange G Loading Dye	Life Technologies	R0631
SYPRO Orange	Merck & Co., Inc.	S5692
SUMO protease	in-house, recombinant	N/A
Acetonitrile	Panreac	721881.1612
Slide-A-Lyzer Mini 7000 MWCO	ThermoFisher	69560
TFA	Merck & Co., Inc.	302031
pOPINS (pOPINS3C was a gift from Ray Owens)	Addgene	Plasmid #41115
Flag-FoxH1 (Flag-FoxH1 was a gift from Stefan Koch)	Addgene	Plasmid #153125
recAf	New England Biolabs	NEB #M0249
QuikChange II Site-Directed Mutagenesis Kit	Qiagen	#200524
QIAquick PCR Purification Kit	Qiagen	#28104
HEK293-T	Merck & Co., Inc.	12022001-DNA-5UG
DMEM HIGH GLUCOSE PYRUVATE	Life Technologies	# 11995073
FBS	Merck & Co., Inc.	F7524
Escherichia coli OverExpress™ C41(DE3) pLysS	Sigma-Aldrich	CAT#CMC0018

Penicillin / Streptomycin	Life Technologies	15140122
L-Glutamine	Life Technologies	25030081
PBS	Panreac	A9162.0100
RIPA Buffer	Merck & Co., Inc.	R0278
DCTM Protein Assay Kit II	BioRad	5000112
ANTI-FLAG® M2 Affinity Gel, purified immunoglobulin	Merck & Co., Inc.	A2220
3x FLAG® Peptide	Merck & Co., Inc.	F4799
Recombinant protein: hFoxH1-FH	This paper	N/A
Recombinant protein: dFoxH1-FH	This paper	N/A
Recombinant protein: hFoxA2-FH	This paper	N/A
Recombinant protein: xFoxH1-FH	This paper	N/A
Recombinant protein: hH3.2	in-house, recombinant	N/A
Recombinant protein: hH2B	in-house, recombinant	N/A
Recombinant protein: hH2A	in-house, recombinant	N/A
Recombinant protein: hH4	in-house, recombinant	N/A

Supplementary Table 5. X-ray data collection and refinement statistics

**Structure**	dFoxH1-GG	dFoxH1-GT	hFoxH1-GG	dFoxH1-TT	dFoxH1-TTAC	hFoxA2-TTAC	hFoxA2-TT	xFoxH1-GG
**PDB entry ID**	7YZ7	7YZA	7YZB	7YZC	7YZD	7YZE	7YZF	7YZG
**Data collection**#^								
Beamline	ALBA BL13	ALBA BL13	ALBA BL13	ALBA BL13	ALBA BL13	ALBA BL13	ALBA BL13	ALBA BL13
Space group	*C*2	*C*2	$P2_1$	$C222_1$	$C222_1$	$C222_1$	$C222_1$	$P2_12_12_1$
a, *b*, *c* (Å)	100.12, 30.09, 76.66	99.39, 29.92, 75.72	36.19, 78.03, 51.88	36.18, 96.56, 153.81	36.24, 96.75, 150.81	46.04, 92.37, 118.80	45.46, 92.70, 116.84	46.30, 78.72, 103.25
α, β, γ (°)	90.00, 108.70, 90.00	90.00, 108.25, 90.00	90.00, 100.46, 90.00	90.00, 90.00, 90.00	90.00, 90.00, 90.00	90.00, 90.00, 90.00	90.00, 90.00, 90.00	90.00, 90.00, 90.00
Resolution (Å)*	47.24-0.98 (1.00-0.98)	46.74-1.18 (1.25-1.18)	51.02-1.47 (1.62-1.47)	76.91-2.17 (2.34-2.17)	46.06-2.13 (2.33-2.13)	46.18-1.98 (2.17-1.98)	58.42-2.18 (2.38-2.18)	62.60-2.82 (3.10-2.82)
R_{meas} (%)	4.6 (136.5)	3.8 (73.4)	11.2 (174.8)	13.8 (125.9)	10.4 (118.3)	4.1 (213.4)	5.6 (177.0)	32.1 (272.1)
R_{pim} (%)	1.8 (68.0)	2.0 (46.9)	2.8 (45.4)	5.5 (48.8)	4.3 (54.8)	1.2 (58.4)	2.4 (74.2)	8.3 (65.8)
$I/\sigma(I)$	17.7 (0.9)	15.4 (1.4)	16.2 (1.5)	7.9 (2.1)	11.7 (1.1)	26.3 (1.2)	19.9 (1.4)	8.2 (1.2)
$CC_{1/2}$	0.999 (0.365)	0.999 (0.554)	0.996 (0.912)	0.995 (0.552)	0.998 (0.373)	0.999 (0.573)	0.998 (0.593)	0.997 (0.484)
Completeness:								
Spherical (%)	89.9 (20.7)	85.1 (32.1)	76.2 (15.1)	82.5 (21.3)	63.7 (14.9)	84.1 (23.2)	69.2 (15.3)	58.0 (10.4)
Ellipsoidal (%)	93.4 (31.5)	89.1 (41.7)	93.1 (50.6)	92.3 (44.7)	90.0 (51.0)	93.9 (53.5)	86.5 (52.2)	90.8 (68.0)
Multiplicity	6.1 (3.7)	3.1 (2.1)	16.8 (14.7)	6.3 (6.4)	5.8 (4.0)	13.1 (13.2)	9.9 (10.3)	14.7 (17.0)
**Refinement**								
Resolution (Å)*	47.23-0.98	46.78-1.18	51.02-1.47	76.91-2.17	46.06-2.13	46.18-1.99	58.42-2.18	62.6-2.82
Number of unique reflections	111,470	54,975	34,784	11,564	9,537	15,058	8,314	5,542
R_{work} / R_{free} (%)	13.2/14.1	14.0/14.8	16.5/19.2	20.8/23.6	21.1/25.5	20.8/21.4	22.2/25.7	20.4/24.8
Protein (aa)	120	121	127	116	112	87	86	121
DNA (bp)	16	16	16	16	16	16	16	16
Number of non-H atoms								
All	2081	1894	1867	1660	1618	1409	1388	1639
Ligand/ion	1	1	1	8	14	1	1	0
Water	360	219	138	46	29	87	5	0
B factors								
Overall	19.3	17.0	41.7	30.4	53.7	68.4	45.6	68.8
Protein	16.8	16.2	40.9	29.0	48.1	58.7	42.1	64.8
DNA	16.4	15.7	42.9	30.9	61.2	79.7	49.3	75.0
Ligand/ion	13.3	11.6	50.2	59.4	57.4	71.9	92.5	NA
Water	31.8	25.2	42.3	47.2	47.5	56.2	60.8	NA

R.M.S.D.								
Bond length (Å)	0.013	0.011	0.007	0.015	0.006	0.009	0.009	0.008
Bond angle (°)	1.45	1.65	1.29	1.74	0.77	1.20	1.17	0.94
Ramachandran:								
Favored (%)	99.2	99.2	100.0	96.4	97.2	98.8	98.8	94.1
Allowed(%)	0.8	0.8	0.0	1.8	2.8	1.2	1.2	5.9
Outliers (%)	0.0	0.0	0.0	1.8	0.0	0.0	0.0	0.0

#Data for the hFoxH1-GG structure come from five merged data sets and for the xFoxH1-GG from three merged data sets. ^Anisotropy correction by STARANISO/autoPROC with the default setting used for the determination of the resolution cutoff [65,75]. *Values in parentheses are for the highest-resolution shell. Resolution cut-off based on paired-refinement protocol implemented in the PDB-REDO server [72]. Space groups are indicated in italics.

Synthetic DNA sequences

(in blue, pOPINS DNA-recombinase sequence.)

hFoxH1 synthetic codon-optimized sequence. (ThermoFisher)

CATCGCGAACAGATCGGTGGTATGGGTCCGAGCAGCGGCTCGCGCTTAGGACCGCCAGAAGCGGAGTCGCCAAGTCAG
CCGCCTAAACGGCGCAAGAAACGTTATCTGCGCCATGATAAGCCGCCGTACACCTATTTGGCCATGATCGCGCTGGTG
ATTCAAGCAGCTCCGTCGCGCCGTTTAAAACTGGCCCAGATCATTCGTCAAGTCCAGGCGGTGTTTCCGTTCTTTCGC
GAAGACTATGAGGGCTGGAAAGATTCCATCCGCCACAATCTGTCTTCGAACCGTTGCTTCCGCAAGGTGCCGAAAGAC
CCTGCAAAGCCGCAGGCCAAAGGCAATTTTTGGGCGGTCGATGTGAGCCTGATTCCAGCTGAAGCGCTCCGTTTACAG
AACACGGCCCTGTGCCGGCGCTGGCAAAATGGAGGTGCGCGTGGCGCCTTCGCGAAAGATCTGGGGCCGTACGTGCTG
CATGGTCGCCCTTATCGTCCGCCAAGTCCGCCTCCGCCACCGAGTGAAGGCTTTAGCATCAAGTCCCTGTTGGGAGGC
AGTGGTGAAGGGGCACCGTGGCCGGGCCTG**TAAAGCTTTCTAGACCATTTAAAC**

dFoxH1 synthetic codon-optimized sequence. (ThermoFisher)

CATCGCGAACAGATCGGTGGTGAACTGCAGGATGGTAACTCGAGTGGCGGAAAAAAGAAAAATTACCAGCGTTATCCG
AAACCACCTTATAGCTACCTGGCCATGATCGCAATGGTCATTCAGAATTCCCCGGAGAAGAAACTTACTTTATCTGAA
ATCCTGAAAGAGATTAGCACGCTCTTTCCGTTCTTTAAGGGGAATTATAAAGGCTGGCGGGATTCAGTTCGCCATAAC
CTGAGCTCCTATGACTGCTTCGTCAAAGTGCTTAAAGATCCAGGCAAGCCTCAGGGTAAAGGGAATTTTTGGACCGTG
GAAGTGAACCGCATTCCGTTGGAGCTGTTGAAACGTCAGAATACAGCAGTGTCGCGTCAAGATGAAACCATCTTCGCG
CAGGACCTGGCGCCGTACATTTTTCAAGGTTAAAGCTTTCTAGACCATTTAAAC

xFoxH1 synthetic codon-optimized sequence. (ThermoFisher)

CATCGCGAACAGATCGGTGGTATGGCAGGAGACCACACACGCAGCCGCAAATCTAAGAAAAAGAATTATCATCGCTAT
TACAAACCGCCATATTCCTACTTAGCTATGATTGCCCTGGTTATCCAGAACTCGCCGGAGAAACGCCTGAAGCTCAGC
CAGATCTTAAAAGAGGTGAGTACCCTGTTCCCGTTCTTTAATGGGGATTATATGGGTTGGAAAGATTCGATCCGCCAC
AACTTGTCTAGCAGTGACTGCTTTAAGAAAATTCTCAAAGATCCTGGAAAGCCGCAAGCCAAAGGTAACTTCTGGACC
GTGGATGTTTCTCGGATTCCGCTGGACGCGATGAAACTGCAGAATACGGCGTTGACCCGCGGTGGCTCAGACTACTTT
GTCCAGGATTTGGCTCCATATATCCTGCATAACTACAAATATGAGCACAATTAAAGCTTTCTAGACCATTTAAAC

hFoxA2 synthetic codon-optimized sequence. (ThermoFisher)

CATCGCGAACAGATCGGTGGTAAAACCTATCGCCGTTCCTATACGCATGCAAAGCCGCCATACAGCTATATCTCTCTC
ATTACCATGGCGATCCAGCAAAGCCCGAACAAAATGCTGACGTTGAGCGAAATTTATCAGTGGATCATGGACTTATTT
CCGTTCTACCGGCAAAATCAGCAACGCTGGCAGAACTCCATTCGTCACTCGCTCTCTTTTAATGATTGTTTCCTGAAA
GTGCCGCGCTCGCCAGACAAGCCGGGCAAAGGTAGTTTTTGGACCCTGCATCCTGATTCGGGCAACATGTTCGAGAAT
GGCTGCTATCTGCGCCGTCAAAAACGCTTTAAGTGCGAAAAACAGCTGGCGTTAAAAGAGGCCGCAGGTGCGGCCGGC
AGCGGGTAAAGCTTTCTAGACCATTTAAAC

Mutation primers

(in red, mutation sites.)

M1: K168A,K164A
Fox_M1KA_Fw GTGCTTAAAGATCCAGGCGCGCCTCAGGGTGCAGGGAATTTTTGGACCGTG
Fox_M1KA_Rv CACGGTCCAAAAATTCCCTGCACCCTGAGGCGCGCCTGGATCTTTAAGCAC

M2:F208A
Fox_M2FA_Fw GACCTGGCGCCGTACATTGCTCAAGGTTAAAGCTTTCTA
Fox_M2FA_Rv TAGAAAGCTTTAACCTTGAGCAATGTACGGCGCCAGGTC

M3:F199A
Fox_M3FA_Fw CGTCAAGATGAAACCATCGCCGCGCAGGACCTGGCGCCG
Fox_M3FA_Rv CGGCGCCAGGTCCTGCGCGGCGATGGTTTCATCTTGACG

M4:K185A
Fox_M4DA_Fw GAAACCATCTTCGCGCAGGCCCTGGCGCCGTACATTTTT
Fox_M4DA_Rv AAAAATGTACGGCGCCAGGGCCTGCGCGAAGATGGTTTC

M4:D202A
Fox_M4KA_Fw ATTCCGTTGGAGCTGTTGGCACGTCAGAATACAGCAGTG
Fox_M4KA_Rv CACTGCTGTATTCTGACGTGCCAACAGCTCCAACGGAAT

M5:Y28F
Fox_Y28F_Fw AGTGGCGGAAAAAAGAAAAATTTCCAGCGTTATCCGAAACCACCT
Fox_Y28F_Rv AGGTGGTTTCGGATAACGCTGGAAATTTTTCTTTTTTCCGCCACT

M6:R30A
Fox_R30A_Fw GGAAAAAAGAAAAATTACCAGGCGTATCCGAAACCACCTTATAGC
Fox_R30A_Rv GCTATAAGGTGGTTTCGGATACGCCTGGTAATTTTTCTTTTTTCC

M7:R30H
Fox_R30H_Fw GGAAAAAAGAAAAATTACCAGCATTATCCGAAACCACCTTATAGC
Fox_R30H_Rv GCTATAAGGTGGTTTCGGATAATGCTGGTAATTTTTCTTTTTTCC

M8:K33N
Fox_K33N_Fw AAAAATTACCAGCGTTATCCGAACCCACCTTATAGCTACCTGGCC
Fox_K33N_Rv GGCCAGGTAGCTATAAGGTGGGTTCGGATAACGCTGGTAATTTTT

M9:ΔWing2
FoxH1h_st_Fw GCACCCTGGCCGGGGCTATGACCACAGAGGAGCGAGTT
FoxH1h_st_Rv AACTCGCTCCTCTGTGGTCATAGCCCCGGCCAGGGTGC

References

1. Aragon E, *et al.* Structural basis for distinct roles of SMAD2 and SMAD3 in FOXH1 pioneer-directed TGF-beta signaling. *Genes Dev* **33**, 1506-1524 (2019).

2. Laskowski RA, Swindells MB. LigPlot+: multiple ligand-protein interaction diagrams for drug discovery. *J Chem Inf Model* **51**, 2778-2786 (2011).

3. Blanchet C, Pasi M, Zakrzewska K, Lavery R. CURVES+ web server for analyzing and visualizing the helical, backbone and groove parameters of nucleic acid structures. *Nucleic Acids Res* **39**, W68-73 (2011).
