# Supplementary figures and images for: Molecular basis for DNA recognition by the maternal pioneer transcription factor FoxH1
Source: Nat Commun. 2022 Nov 26;13:7279. doi: 10.1038/s41467-022-34925-y (PMC9701222; doi:10.1038/s41467-022-34925-y)

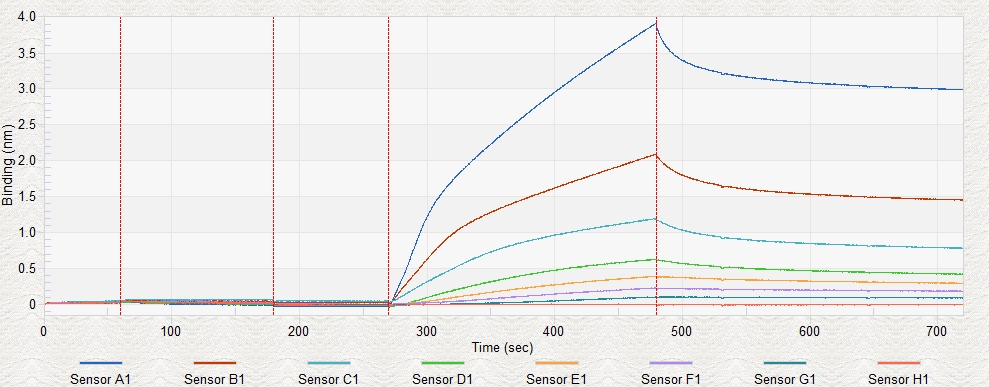

Supplement: Supplementary file 4 — Source Data [file 41467_2022_34925_MOESM4_ESM.zip › Sourcedata/BLIS/FoxH1/WT/GSC/DNA/220309_P1_Assay_1.jpg]

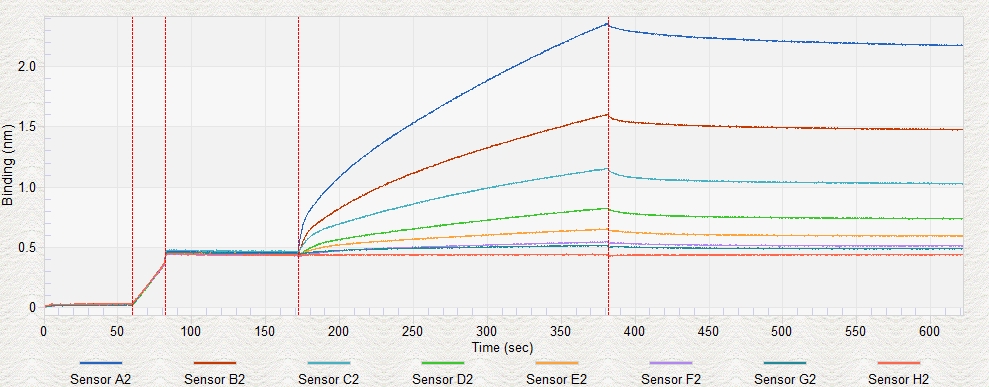

Supplement: Supplementary file 4 — Source Data [file 41467_2022_34925_MOESM4_ESM.zip › Sourcedata/BLIS/FoxH1/WT/GSC/NCP/220309_P1_Assay_2.jpg]

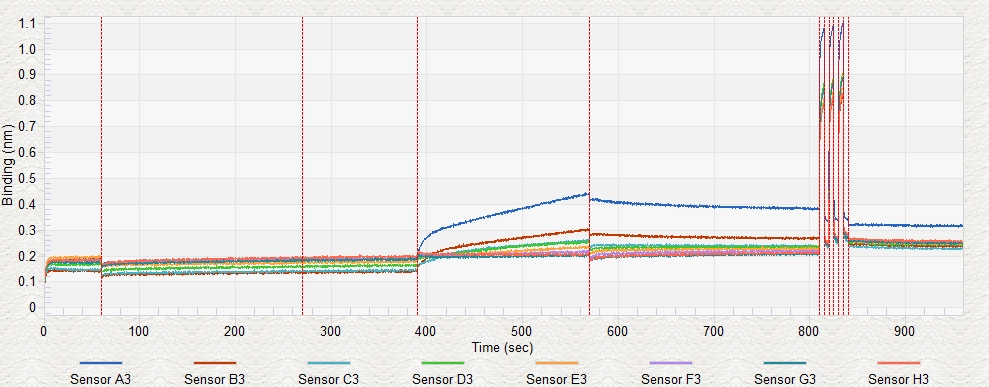

Supplement: Supplementary file 4 — Source Data [file 41467_2022_34925_MOESM4_ESM.zip › Sourcedata/BLIS/FoxH1/WT/601/-6/DNA/220913_Assay_3.jpg]

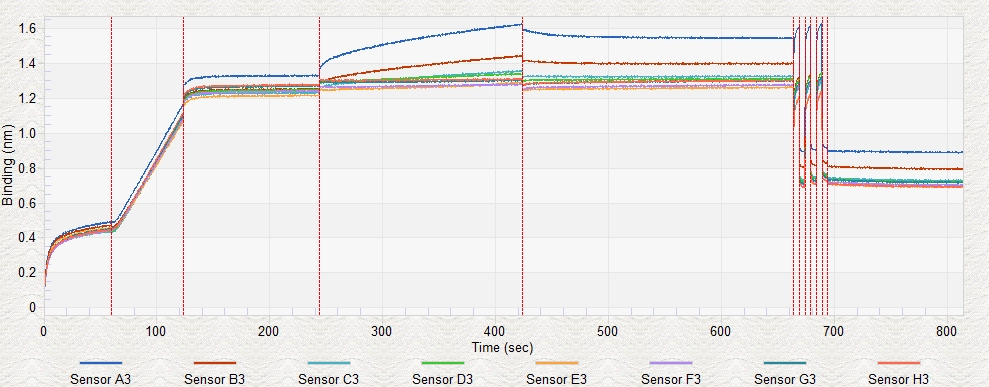

Supplement: Supplementary file 4 — Source Data [file 41467_2022_34925_MOESM4_ESM.zip › Sourcedata/BLIS/FoxH1/WT/601/-6/NCP/220913_Assay_3.jpg]

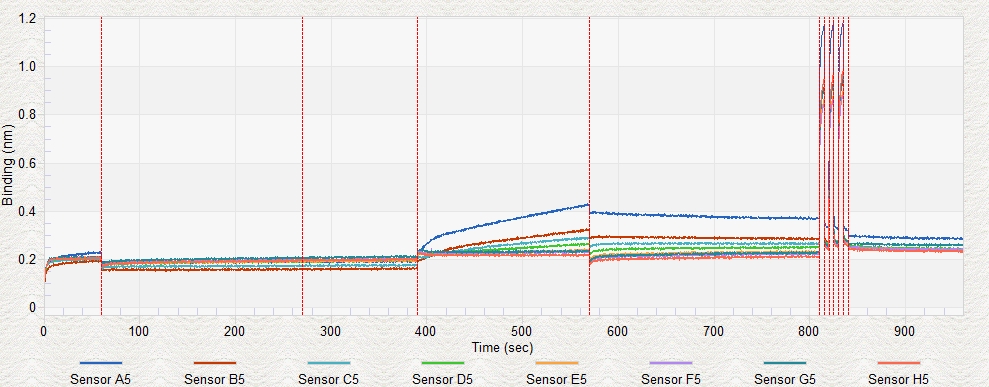

Supplement: Supplementary file 4 — Source Data [file 41467_2022_34925_MOESM4_ESM.zip › Sourcedata/BLIS/FoxH1/WT/601/+0.5/DNA/220913_Assay_2.jpg]

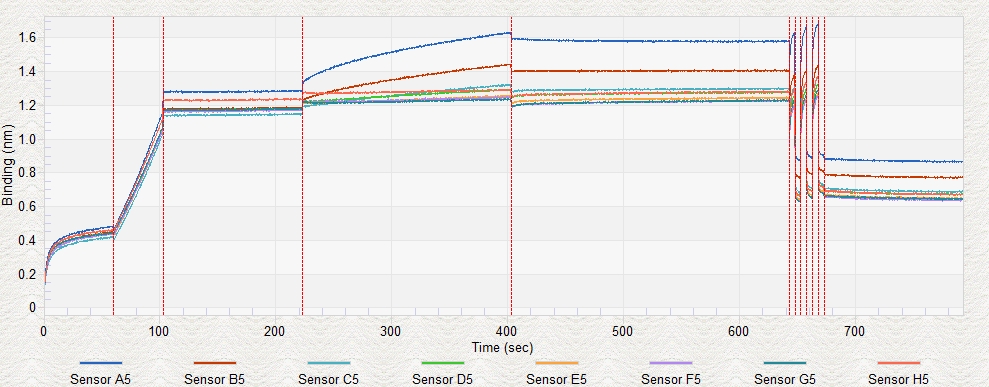

Supplement: Supplementary file 4 — Source Data [file 41467_2022_34925_MOESM4_ESM.zip › Sourcedata/BLIS/FoxH1/WT/601/+0.5/NCP/220913_Assay_2.jpg]

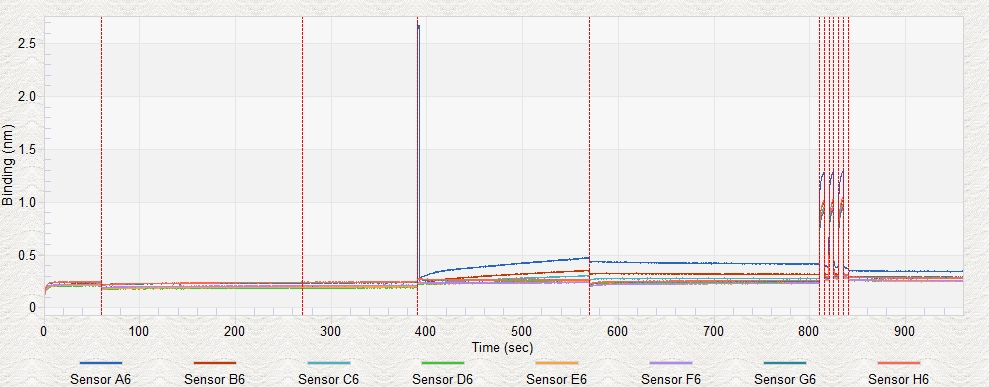

Supplement: Supplementary file 4 — Source Data [file 41467_2022_34925_MOESM4_ESM.zip › Sourcedata/BLIS/FoxH1/WT/601/+2.5/DNA/220913_Assay_3.jpg]

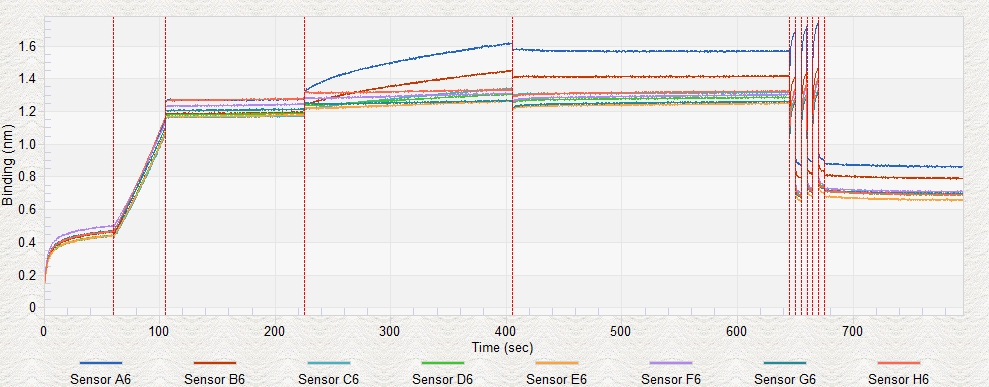

Supplement: Supplementary file 4 — Source Data [file 41467_2022_34925_MOESM4_ESM.zip › Sourcedata/BLIS/FoxH1/WT/601/+2.5/NCP/220913_Assay_3.jpg]

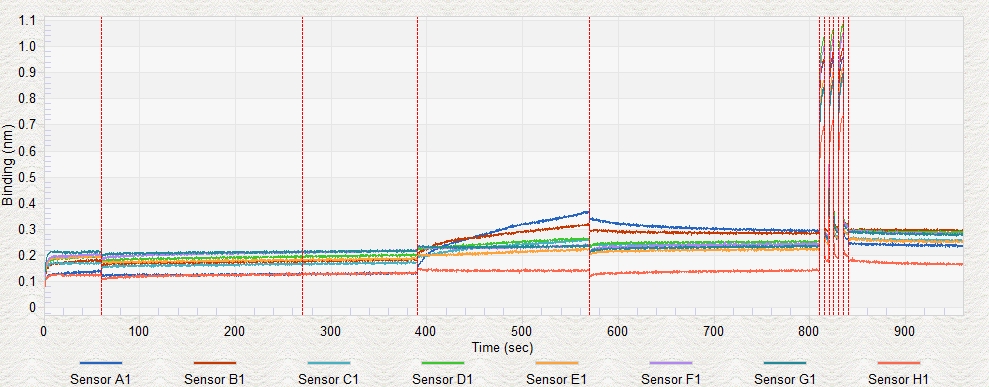

Supplement: Supplementary file 4 — Source Data [file 41467_2022_34925_MOESM4_ESM.zip › Sourcedata/BLIS/FoxH1/WT/601/+6/DNA/220913_Assay_1.jpg]

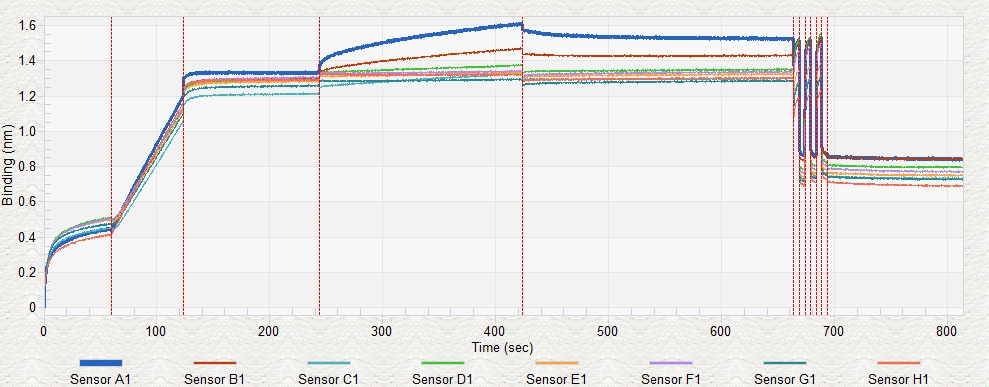

Supplement: Supplementary file 4 — Source Data [file 41467_2022_34925_MOESM4_ESM.zip › Sourcedata/BLIS/FoxH1/WT/601/+6/NCP/220913_Assay_1.jpg]

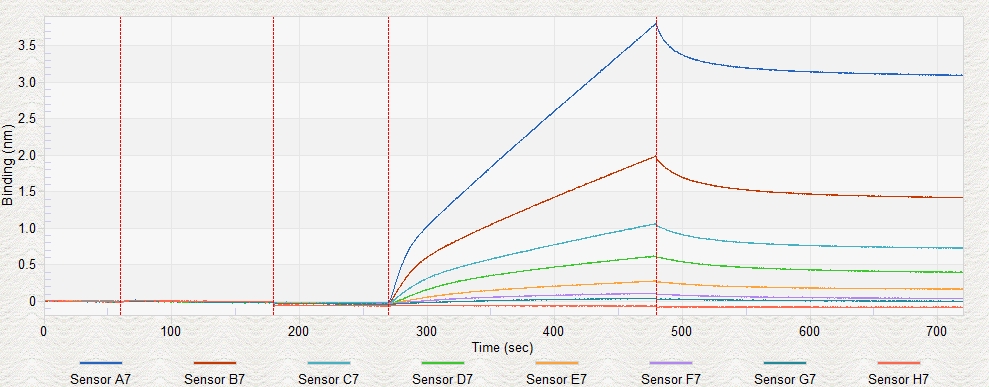

Supplement: Supplementary file 4 — Source Data [file 41467_2022_34925_MOESM4_ESM.zip › Sourcedata/BLIS/FoxH1/R30H/GSC/DNA/220309_P2_Assay_1.jpg]

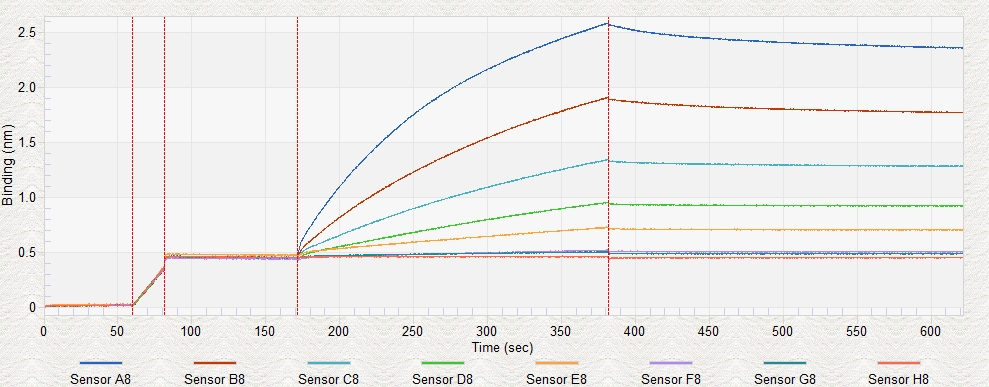

Supplement: Supplementary file 4 — Source Data [file 41467_2022_34925_MOESM4_ESM.zip › Sourcedata/BLIS/FoxH1/R30H/GSC/NCP/220309_P2_Assay_2.jpg]

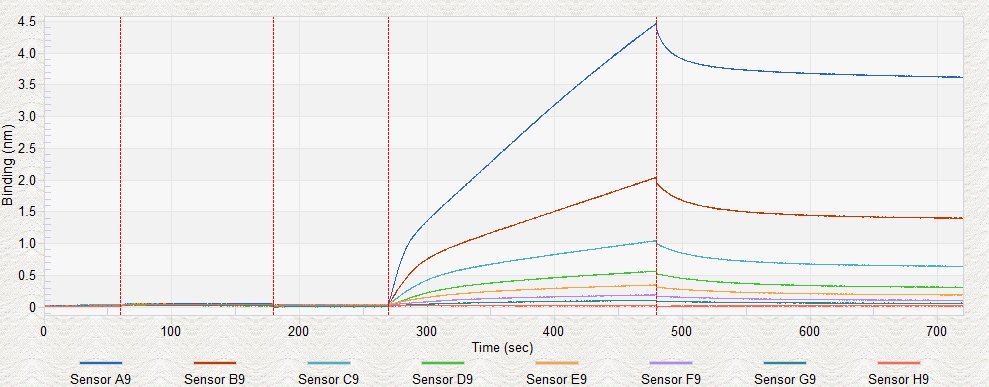

Supplement: Supplementary file 4 — Source Data [file 41467_2022_34925_MOESM4_ESM.zip › Sourcedata/BLIS/FoxH1/K33N/GSC/DNA/220309_P2_Assay_3.jpg]

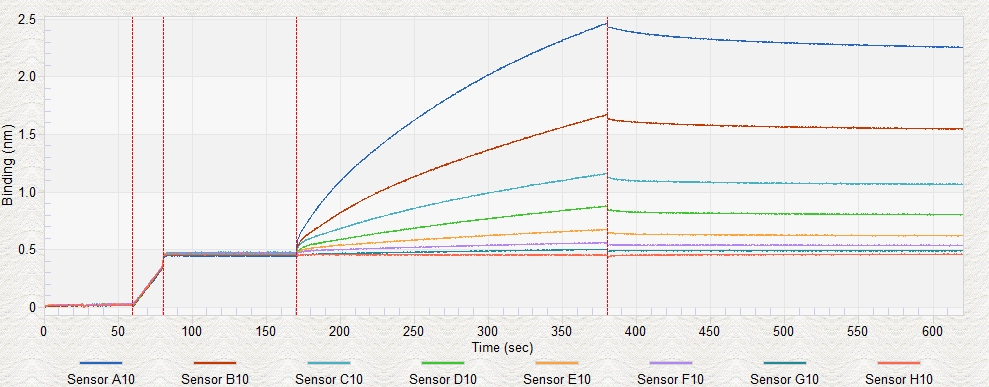

Supplement: Supplementary file 4 — Source Data [file 41467_2022_34925_MOESM4_ESM.zip › Sourcedata/BLIS/FoxH1/K33N/GSC/NCP/220309_P2_Assay_4.jpg]

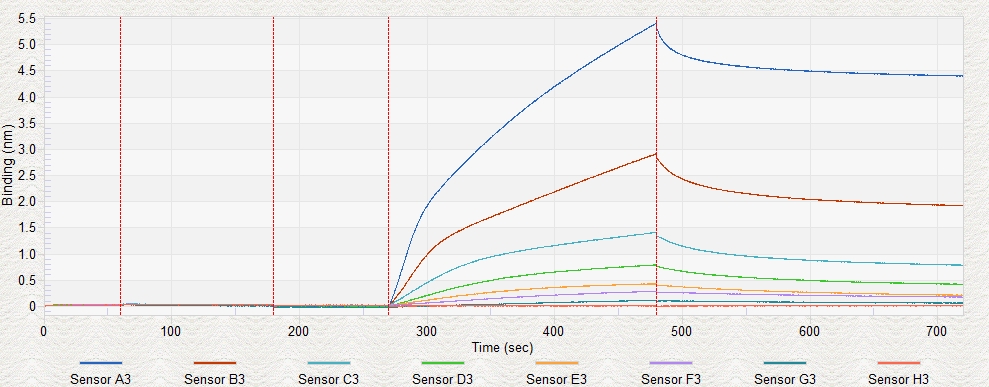

Supplement: Supplementary file 4 — Source Data [file 41467_2022_34925_MOESM4_ESM.zip › Sourcedata/BLIS/FoxH1/Y28F/GSC/DNA/220309_P1_Assay_3.jpg]

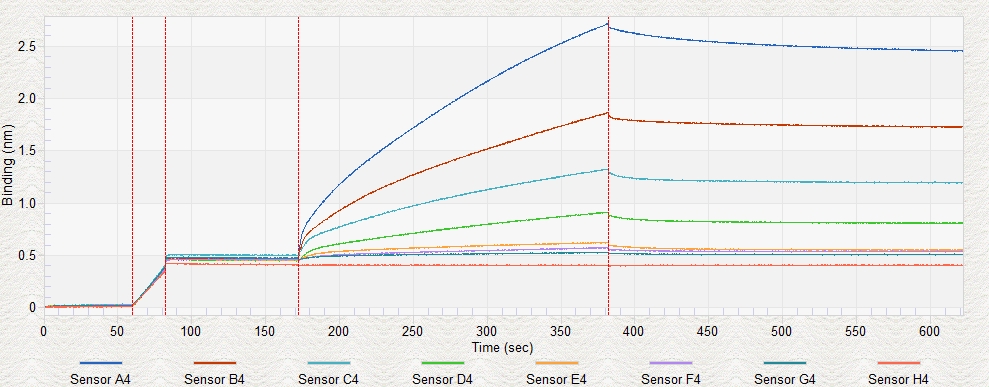

Supplement: Supplementary file 4 — Source Data [file 41467_2022_34925_MOESM4_ESM.zip › Sourcedata/BLIS/FoxH1/Y28F/GSC/NCP/220309_P1_Assay_4.jpg]

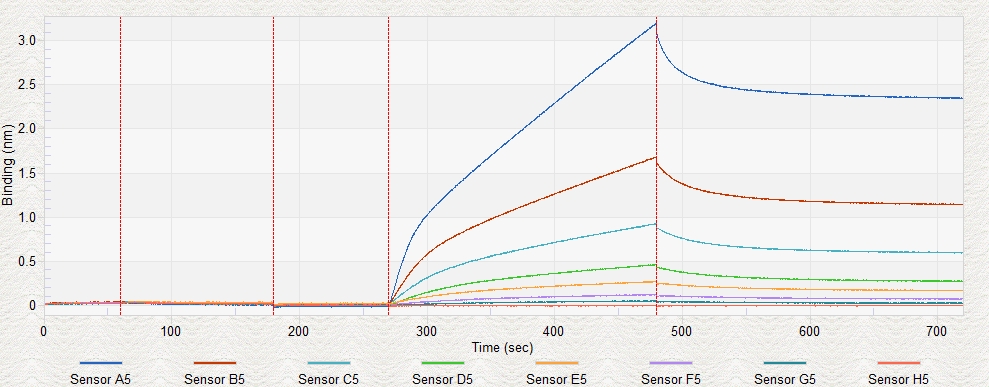

Supplement: Supplementary file 4 — Source Data [file 41467_2022_34925_MOESM4_ESM.zip › Sourcedata/BLIS/FoxH1/R30A/GSC/DNA/220309_P1_Assay_5.jpg]

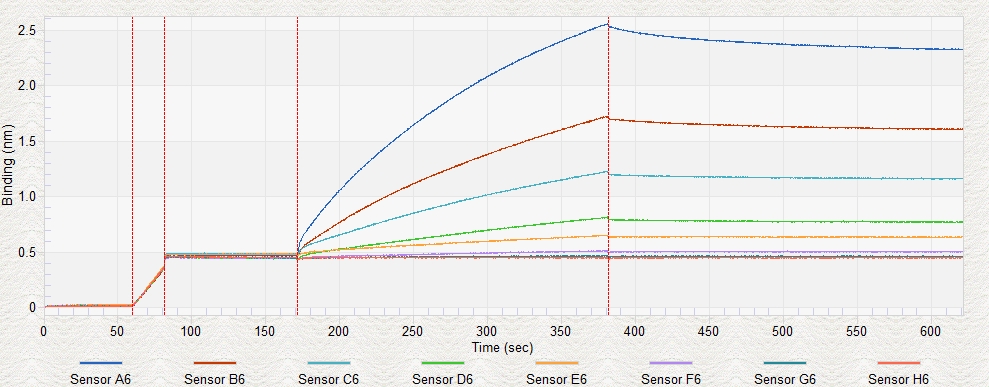

Supplement: Supplementary file 4 — Source Data [file 41467_2022_34925_MOESM4_ESM.zip › Sourcedata/BLIS/FoxH1/R30A/GSC/NCP/220309_P1_Assay_6.jpg]

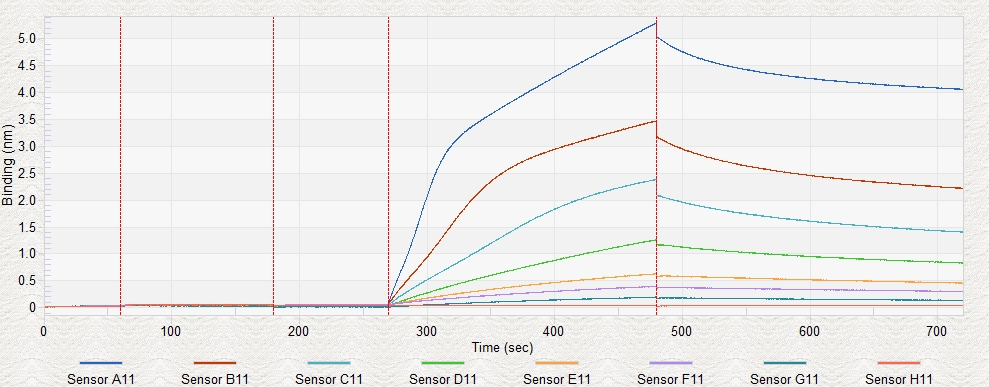

Supplement: Supplementary file 4 — Source Data [file 41467_2022_34925_MOESM4_ESM.zip › Sourcedata/BLIS/FoxA2/GSC/DNA/220309_P2_Assay_5.jpg]

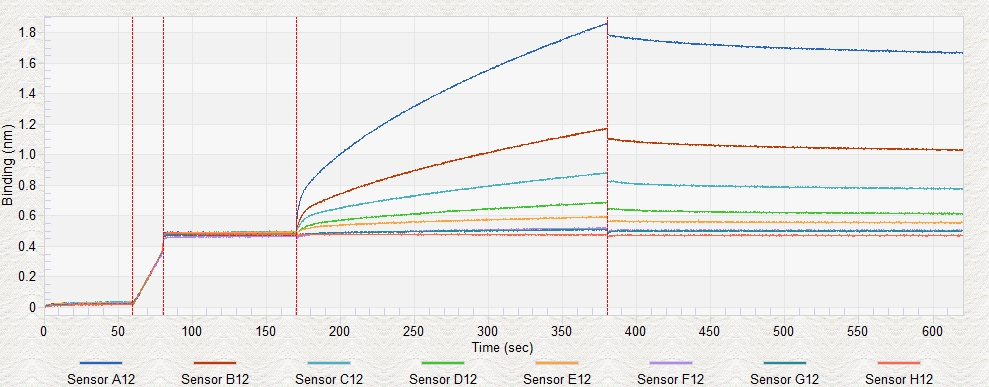

Supplement: Supplementary file 4 — Source Data [file 41467_2022_34925_MOESM4_ESM.zip › Sourcedata/BLIS/FoxA2/GSC/NCP/220309_P2_Assay_6.jpg]
